# Biomechanical characterization of firefighters running under different rescue tasks

**DOI:** 10.1038/s41598-024-52440-6

**Published:** 2024-01-20

**Authors:** Xinxin Zhang, Haili Feng, Xindai Liu, Pincao Gao, Peng Guo, Shenghui Tang, Xinhe Nie, Tianjin Feng, Weiguo Liu

**Affiliations:** 1 College of Physical Education and Health, Guangxi Normal University, Guilin, 541004 China; 2 College of International Culture and Education, Guangxi Normal University, Guilin, 541004 China; 3 Guilin Yanshan District Fire Rescue Brigade, Guilin, 541004 China; 4 College of Cotinuing Education, Guilin Medical University, Guilin, 541004 China

**Keywords:** Biotechnology, Health care

## Abstract

The biomechanical characteristics of runs in firefighters with different rescue tasks are unclear. This study aimed to explore the biomechanical characteristics of firefighters running in different rescue tasks and provide theoretical and practical references for firefighter training and occupational injury prevention. Eighteen professional healthy male firefighters were randomly selected as participants and tested running on different rescue tasks: wearing firefighting protective clothing (FPC), FPC+carrying a gas can (20 kg, FPC+ C), and FPC+carrying a mannequin (60 kg, FPC+M). Eight Qualisys infrared cameras and an AMTI 3D force measurement platform were used for the participant's acquisition of lower limb kinematic/kinetic data. The results showed that gait velocity and stride length of the FPC+GC and FPC+ M rescue tasks were significantly decreased compared to the FPC rescue task, while the support time was significantly increased. Compared to the FPC rescue task, the FPC+GC rescue task showed significant decreases in vertical ground reaction force (vGRF), minimum ankle dorsiflexion angle, and the maximum ankle plantarflexion power. In contrast, the FPC+M rescue task demonstrated significant increases in ankle range of motion, maximum hip extension angle, minimum knee flexion angle, maximum ankle dorsiflexion angle, maximum hip extension moment, maximum knee flexion moment, maximum hip flexion power, and hip and knee stiffness while exhibiting significant decreases in minimum ankle dorsiflexion angle. Compared to the FPC+ GC rescue task, the FPC+M rescue task exhibited significant increases in the maximum hip extension angle, minimum knee flexion angle, maximum ankle dorsiflexion angle, maximum hip flexion moment, maximum hip extension moment, maximum knee flexion moment, maximum ankle plantarflexion moment, maximum hip flexion power, maximum ankle dorsiflexion power, hip stiffness, and vGRF. Conversely, it showed significant decreases in the maximum knee flexion power. In conclusion, compared to the FPC rescue task, the FPC+GC and FPC+M rescue tasks altered the firefighter's gait performance, as evidenced by decreased gait velocity and stride length and increased support time. FPC+M rescue task would increase firefighter's risk of hip and knee injuries. Therefore, we suggest firefighters increase their strength training of the trunk, hip, and knee joint muscles as part of their daily training programs under large weight load status (60 kg and above) to reduce injury risk during rescue tasks.

## Introduction

Firefighting has a greater injury rate than most other professions^1,2^. Over 80,000 firefighter injuries occur annually worldwide, most occurring on the fire ground^3^. Due to the special nature of work, firefighters must wear firefighting protective clothing (FPC) to ensure their safety during the mission. However, FPC has some negative impacts while protecting firefighters. Previous studies have shown^4,5^ that FPC could significantly increase firefighters' physiological burden and reduce their physical flexibility. Consequently, enhancing the rescue performance of firefighters while maintaining their safety has emerged as an essential focus in prior studies. However, most of them focus on the scientific investigation of physical conditions, such as the improved design of FPC^1,3,6–9^ and boots^10–12^. In fact, in fire rescue situations, the rational use of firefighters' individualized behaviors, such as motor performance and control, is crucial for improving firefighters' rescue performance.

The three primary characteristics of fire rescue tasks are complexity, unpredictability, and time sensitivity. The goal of fire rescue is to complete rescue tasks swiftly and safely. In most rescue situations, firefighters must move quickly to perform their duties despite the significant obstacles posed by the size and weight of the rescue equipment. Moreover, the objects that firefighters must carry are usually irregular in shape and heavy, which affects their biomechanics during rescue^13^ and could reduce their efficiency and balance^14^. To improve their rescue abilities, firefighters often engage in complex daily training tasks, such as carrying heavy rescue equipment or objects like gas cans and mannequins while running^15^. This training not only tests firefighters' physical abilities and endurance but also requires them to learn the proper way to carry loads and move rhythmically, increase the efficiency of movement, and avoid wasting oxygen consumption, thus jeopardizing the life in the fire rescue. Although this training significantly enhances firefighters' rescue abilities, it has also increased the percentage of firefighters injured as a result of the training^2^. To improve the rescue ability of firefighters, in addition to increasing training, two aspects of optimization of rescue technique movements and prevention of sports injuries could be used. While the analysis based on kinematic indicators such as joint angles could help firefighters to optimize the movements of rescue techniques, the analysis based on kinetic indicators such as joint moments and stiffness could help firefighters to identify the injury risks of fire rescue techniques^16^.

Therefore, to better serve fire rescue training, improve rescue quality and reduce the risk of rescue injuries. In this study, three different conditions of rescue tasks were selected for biomechanical analysis, aiming to explore the running biomechanical characteristics of different rescue tasks and compare their intrinsic differences to provide theoretical and practical references for the optimization of rescue techniques and injury prevention in firefighters' daily training and actual rescue.

## Methods

### Participants

Eighteen professional healthy male firefighters were randomly selected as participants (age: 25.5 ± 6.7 years, height: 171.9 ± 5.9 cm, weight: 77.62 ± 3.42 kg, years of service: 6.2 ± 2.1 years), and all participants were running with heel strike technique (It was learned for through inquiries). Before the test, participants were asked to perform four kick soccer tests according to their habits to determine the participant's dominant leg (the kicking leg utilized multiple times). The results showed that all participants' dominant leg was the right. The inclusion criteria for firefighters were no history of injury or medication use within the last year. All participants were given written informed consent to this experiment, which conformed to the standards set by the Declaration of Helsinki and was approved by the ethics committee of Guangxi Normal University.

### Apparatus and measurement

Eight Qualisys infrared cameras (Oqus600, Sweden) with 14 mm marker spheres were used for participant kinematic data acquisition with a sampling frequency of 200 Hz. The mark spheres were set up in reference to the Helen Hayes model to mark 36 bony landmarks in the lower extremity^17^. AMTI 3D force measurement platform (AMTI, Watertown, MA) was used for participant kinetic data acquisition with a sampling frequency of 1000 Hz. A digital-to-analogue converter synchronized the infrared motion capture system with the force platform.

**Figure 1:**
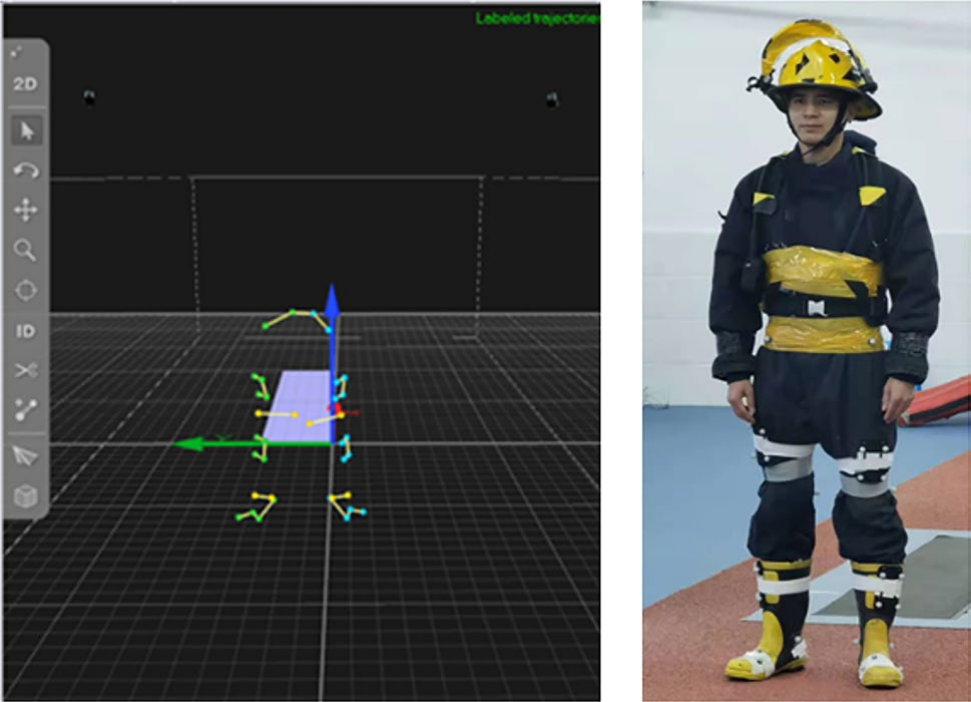
Static data acquisition.

**Figure 2:**
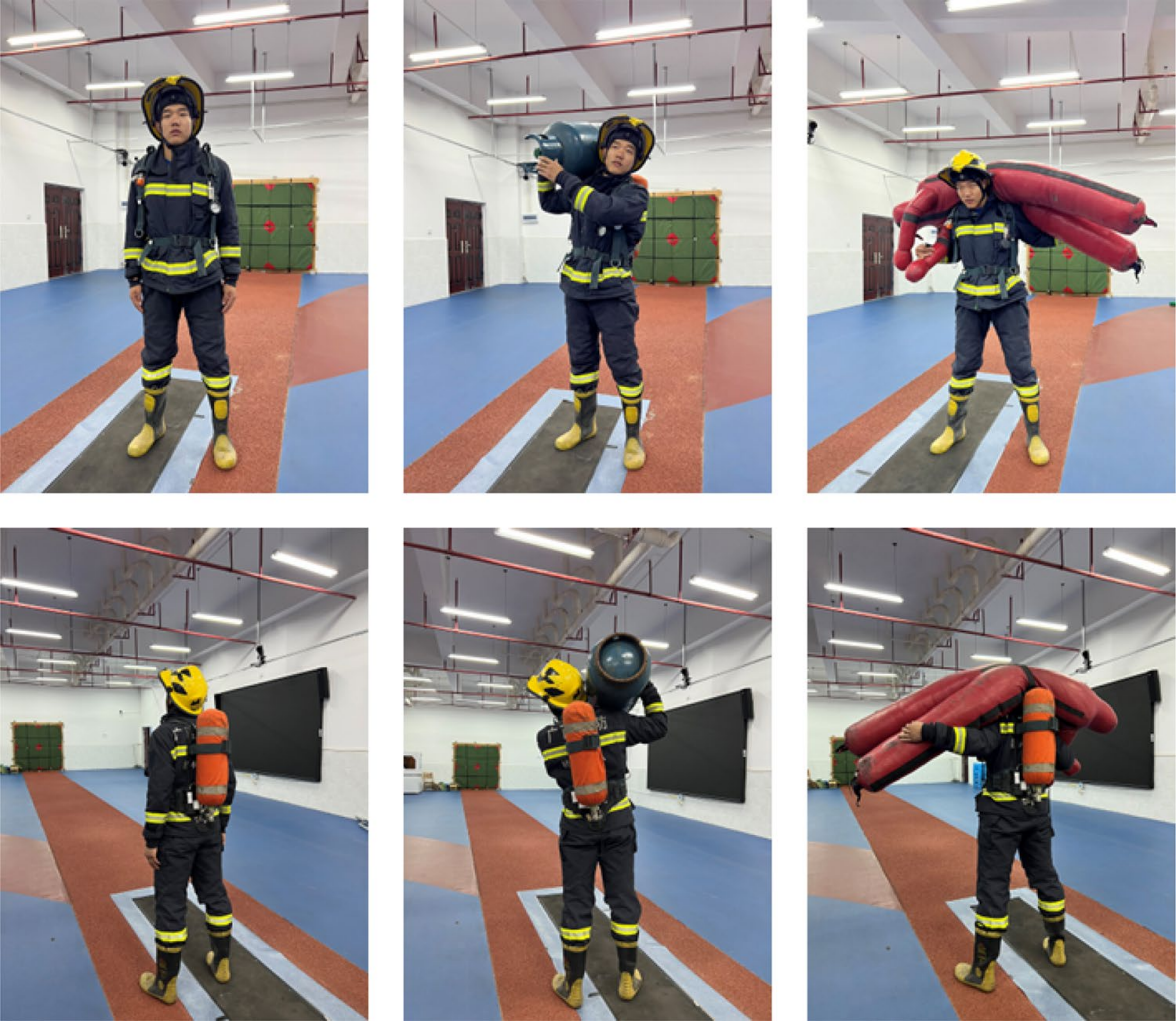
Rescue tasks.

After running a warm-up (self-paced, 400 m), all participants were asked to wear the same type of FPC for static lower extremity data acquisition for post-data modelling and analysis (Fig. 1). Dynamic data acquisition after static data acquisition is completed. Before beginning dynamic data acquisition, participants were required to complete three natural running trials along a specified 12 m linear walkway to identify the starting position of each running and to maintain the regularity of the experiment. Dynamic data acquisition was carried out according to different rescue tasks, which mainly included wearing an FPC, wearing an FPC and carrying a gas can (20 kg, FPC+GC), and wearing an FPC and carrying a mannequin (60 kg, FPC+M). These tasks were selected with the assistance of the Fire Department’s Training Officer and were identified as typical tasks performed by this Department (Fig. 2). During data acquisition, participants were instructed to execute the rescue task at their fastest running speed while allowing appropriate rest time. Each participant was tested three times under each rescue task, and valid data were collected. In order to avoid the randomness of the experimental results, the final presentation of the parameter data of this study is the average of three valid data, and one valid data was regarded when the following conditions were met. Throughout the acquisition, the participant's foot did not step off the edge of the force platform, and no marker spheres were lost.

### Experimental parameters and data processing

Running could be divided into two periods: stance and swing. The period selected in this study was the stance period of the dominant leg, defined as the moment when the heel touches the force platform (vertical ground reaction force, vGRF > 10N) to the moment when its toe leaves the force platform (vGRF < 10N)^16^.

A 3D model of the human lower extremity was constructed using the biomechanical analysis software Visual 3D (C-Motion Inc., Bethesda, MD, USA). The kinematic data of the stance period were filtered using a Butterworth fourth-order low-pass digital filter at a cutoff frequency of 14 Hz^18^. The kinematic parameters mainly included step width, gait velocity (average velocity on the force plate), cadence, stride length, support time, hip, knee, and ankle joint angles and range of motion (ROM) in the sagittal plane during the stance period. Joint angle and ROM parameters were defined as follows: Hip joint angle: the angle between pelvic and thigh coordinates; positive values indicate hip flexion and negative values indicate hip extension. Knee joint angle: The angle between calf and thigh coordinates; positive values indicate knee extension and negative values indicate knee flexion. Ankle joint angle: The angle between calf and foot coordinates; positive values indicate dorsiflexion and negative values indicate plantarflexion. ROM: The absolute value of the maximum joint flexion angle minus the maximum joint extension angle (for the ankle joint, it is the maximum dorsiflexion angle minus the maximum plantarflexion angle).

All kinetic signals were filtered through a fourth-order Butterworth digital filter at cutoff frequencies of 50 Hz^16^. To eliminate the effect of body weight on the results, all kinetic parameters were standardized by dividing by weight. The parameters include hip, knee, and ankle moments, power, stiffness, and maximum vGRF in the sagittal plane during the stance period, and the kinetic parameters used are divided by the individual weight of the participant for standardization. Among all the kinetic parameters, vGRF was measured and exported by Visual 3D software, and other relevant parameters were defined as follows. Joint moment: the definition is the same as the joint angle. Joint power^16^: product of the joint moment and joint angular velocity. Joint stiffness^19^: calculated by the ratio of the joint moment to joint angular displacement.

### Statistical analysis

Each kinematic and kinetic parameter was tested for normal distribution. If it followed the normal distribution, one-way repeated measures ANOVA (posthoc test: Bonferroni) was used for statistical analysis, and if it did not follow the normal distribution, nonparametric tests (Mann–Whitney U-test and Kruskal–Wallis test) were performed, and the statistical significance level was set at 0.05. Data were expressed as mean ± standard deviation (Mean ± SD), and all data in this study were processed using SPSS 21.0 software (IBMS, NY, USA). Based on previous studies^20^, partial eta-squared $(\eta_{{\text{p}}}^{2} )$ was used to report effect sizes, specifying 0.1–0.25 as a small effect size, 0.25–0.4 as a medium effect size, and greater than 0.4 as a large effect size.

### Institutional review board

Participants gave written informed consent to this experiment which conformed to the standards set by the Declaration of Helsinki and was approved by the ethics committee of Guangxi Normal University (20230419001).

### Informed consent

Informed consent was obtained from all subjects and/or their legal guardian(s) for both study participation AND publication of identifying information/images in an online open-access publication.

## Results

### Spatiotemporal gait parameters

The results showed that compared with the FPC group, the gait velocity (FPC+GC: *p* = 0.002, ${{{\upeta}}}_{p}^{2}$=0.44, FPC+M: *p* = 0.013, ${{{\upeta}}}_{p}^{2}$=0.40) and stride length (FPC+GC: *p* = 0.005, ${{{\upeta}}}_{p}^{2}$=0.31, FPC+M: *p* = 0.001, ${{{\upeta}}}_{p}^{2}$=0.29) were significantly decreased, and the support time was significantly increased in the FPC+GC (*p* = 0.006, ${{{\upeta}}}_{p}^{2}$=0.26) and FPC+M group (*p* = 0.02, ${{{\upeta}}}_{p}^{2}$=0.28); compared with the FPC+GC rescue task, the support time of the FPC+M rescue task was significantly increased (*p* = 0.002, ${{{\upeta}}}_{p}^{2}$=0.44); the step width (*p* = 0.17, ${{{\upeta}}}_{p}^{2}$=0.16) and cadence (*p* = 0.20, ${{{\upeta}}}_{p}^{2}$=0.20) did not show significant differences between the groups. The specific results are shown in Table 1.

**Table 1:** Spatio-temporal gait parameters of different rescue tasks during the stance period (N = 18).

Gait parameters	FPC	FPC+GC	FPC+M
Step width (m)	0.06 ± 0.05	0.04 ± 0.04	0.06 ± 0.06
Gait velocity (m/s)	3.84 ± 0.55	3.24 ± 0.34*	3.13 ± 0.27^#^
Cadence (step/min)	181.54 ± 26.56	182.90 ± 11.75	177.68 ± 16.17
Stride length (m)	2.57 ± 0.28	2.15 ± 0.17*	2.09 ± 0.12^#^
Support time (s)	0.23 ± 0.03	0.29 ± 0.02*	0.33 ± 0.04^#%^

“*”, “#” and “%” indicate significant differences between FPC group and FPC+GC group, FPC group and FPC+M group, and FPC+GC group and FPC+M group respectively.

### Kinematic and kinetic parameters

**Figure 3:**
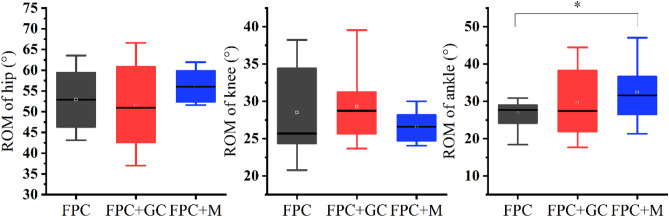
Range of joint motion. “*” indicate significant differences between FPC group and FPC+M group.

**Figure 4:**
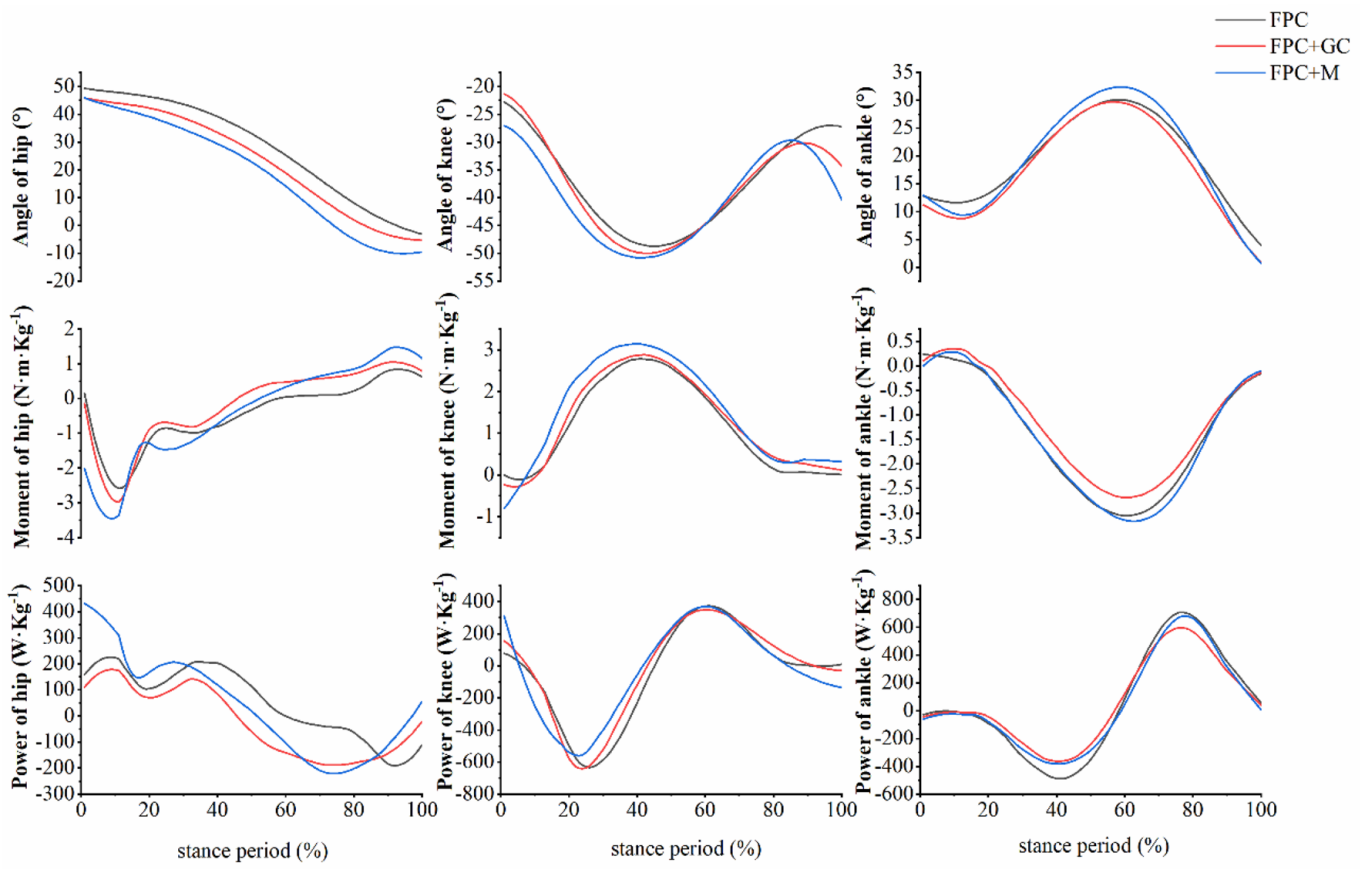
Average trends in lower limb joint parameters (N = 18).

The results of the joint angle showed that compared with the FPC group, in the FPC+GC group, the minimum ankle dorsiflexion angle was significantly decreased (*p* < 0.001, ${{{\upeta}}}_{p}^{2}$=0.33). Compared with the FPC group, in the FPC+M group, the minimum ankle dorsiflexion angle was significantly decreased (*p* = 0.049, ${{{\upeta}}}_{p}^{2}$=0.27); the ROM of the ankle (*p* = 0.01, ${{{\upeta}}}_{p}^{2}$=0.42), the maximum hip extension angle (*p* = 0.04, ${{{\upeta}}}_{p}^{2}$=0.28), the minimum knee flexion angle (*p* < 0.001, ${{{\upeta}}}_{p}^{2}$=0.35), and the maximum ankle dorsiflexion angle (*p* = 0.03 ${{{\upeta}}}_{p}^{2}$=0.48) was significantly increased. Compared to the FPC+GC group, in the FPC+M group, the maximum hip extension angle (*p* = 0.03 ${{{\upeta}}}_{p}^{2}$=0.42), the minimum knee flexion angle (*p* = 0.02, ${{{\upeta}}}_{p}^{2}$=0.46), and the maximum ankle dorsiflexion angle was significantly increased (*p* = 0.01, ${{{\upeta}}}_{p}^{2}$=0.29); the rest of the parameters were not significantly different. The specific results are shown in Figs. 3 and 4 and Table 2.

**Table 2:** The joint angle of different rescue tasks during the stance period (N = 18).

Parameters	FPC	FPC+GC	FPC+M
Maximum hip flexion angle (°)	49.58 ± 7.24	45.98 ± 5.59	45.94 ± 5.04
Maximum hip extension angle (°)	3.32 ± 3.28	5.59 ± 5.48	10.19 ± 5.38^#%^
Minimum knee flexion angle (°)	20.36 ± 6.06	20.81 ± 6.00	24.55 ± 4.46^#%^
Maximum knee flexion angle (°)	48.85 ± 5.60	50.13 ± 5.82	51.12 ± 4.47
Maximum ankle dorsiflexion angle (°)	30.18 ± 4.87	29.89 ± 4.99	32.55 ± 2.99^#%^
Minimum ankle dorsiflexion angle (°)	3.26 ± 6.94	0.23 ± 6.08*	0.14 ± 6.68^#^

“*”, “#” and “%” indicate significant differences between FPC group and FPC+GC group, FPC group and FPC+M group, and FPC+GC group and FPC+M group respectively.

The joint moment and power results showed that compared with the FPC group, the maximum ankle plantarflexion power (*p* = 0.02, ${{{\upeta}}}_{p}^{2}$=0.44) was significantly decreased in the FPC+GC group; the maximum hip extension moment (p = 0.04, ${{{\upeta}}}_{p}^{2}$=0.37), the maximum knee flexion moment (*p* = 0.01, ${{{\upeta}}}_{p}^{2}$=0.26), and the maximum hip flexion power (*p* = 0.02, ${{{\upeta}}}_{p}^{2}$=0.36) was significantly increased in the FPC+M group. Compared with the FPC+GC group, in the FPC+M group, the maximum hip flexion moment (*p* < 0.001, ${{{\upeta}}}_{p}^{2}$=0.47), the maximum hip extension moment (*p* = 0.049, ${{{\upeta}}}_{p}^{2}$=0.33), the maximum knee flexion moment (*p* < 0.001, ${{{\upeta}}}_{p}^{2}$=0.44), the maximum ankle plantarflexion moment (*p* = 0.04, ${{{\upeta}}}_{p}^{2}$=0.47), the maximum hip flexion power (p = 0.03, ${{{\upeta}}}_{p}^{2}$=0.34), and the maximum ankle dorsiflexion power (*p* < 0.001, ${{{\upeta}}}_{p}^{2}$=0.29) was significantly increased; the maximum knee flexion power (*p* = 0.02, ${{{\upeta}}}_{p}^{2}$=0.36) was significantly decreased; the remaining parameters were not significantly different. The specific results are shown in Fig. 4 and Table 3.

**Table 3:** Joint moment and power of rescue tasks during the stance period (N = 18).

Parameters	FPC	FPC+GC	FPC+M
Maximum hip flexion moment (N m kg^−1^)	1.39 ± 0.47	1.27 ± 0.28	1.76 ± 0.55^%^
Maximum hip extension moment (N m kg^−1^)	4.29 ± 1.34	4.34 ± 0.86	6.42 ± 1.32^#%^
Maximum knee extension moment (N m kg^−1^)	2.65 ± 0.72	2.79 ± 0.92	2.97 ± 1.21
Maximum knee flexion moment (N m kg^−1^)	0.94 ± 0.70	0.94 ± 0.66	1.41 ± 0.68^#%^
Maximum ankle dorsiflexion moment (N m kg^−1^)	0.84 ± 0.41	0.82 ± 0.35	1.11 ± 0.55
Maximum ankle plantarflexion moment (N m kg^−1^)	3.13 ± 0.77	2.74 ± 0.54	3.23 ± 0.55^%^
Maximum hip flexion power (W kg^−1^)	407.75 ± 287.60	344.21 ± 187.74	739.02 ± 330.10^#%^
Maximum hip extension power (W kg^−1^)	271.72 ± 105.23	240.39 ± 127.35	281.94 ± 201.61
Maximum knee extension power (W kg^−1^)	439.43 ± 218.28	482.66 ± 177.32	433.98 ± 210.79
Maximum knee flexion power (W kg^−1^)	668.67 ± 269.17	755.28 ± 334.52	591.57 ± 345.02^%^
Maximum ankle dorsiflexion power (W kg^−1^)	735.72 ± 322.01	624.52 ± 272.29	727.85 ± 176.01^%^
Maximum ankle plantarflexion power (W kg^−1^)	555.92 ± 208.47	397.97 ± 191.49*	443.99 ± 77.02

“*”, “#” and “%” indicate significant differences between FPC group and FPC+GC group, FPC group and FPC+M group, and FPC+GC group and FPC+M group respectively.

**Figure 5:**
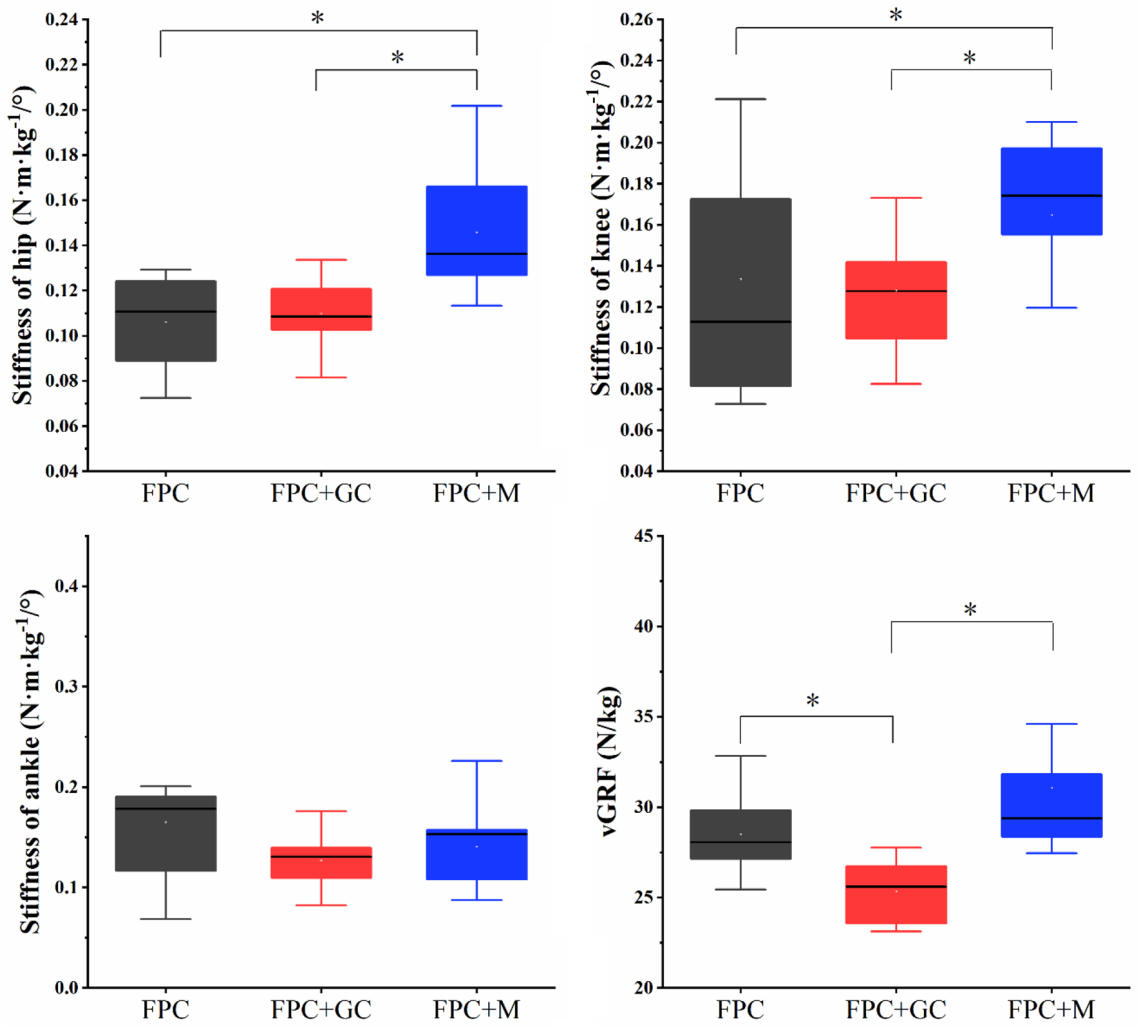
Joint stiffness and vGRF. “*” indicate significant differences between groups.

The joint stiffness and vGRF showed that the hip and knee stiffness significantly increased in the FPC+M groups compared to the FPC (hip: *p* = 0.03, ${{{\upeta}}}_{p}^{2}$=0.39, knee: *p* = 0.001, ${{{\upeta}}}_{p}^{2}$=0.56) and FPC+GC groups (hip: *p* = 0.005, ${{{\upeta}}}_{p}^{2}$=0.33, knee: *p* = 0.04, ${{{\upeta}}}_{p}^{2}$=0.29). Compared with the FPC+GC group, vGRF was significantly increased in the FPC group (*p* = 0.049, ${{{\upeta}}}_{p}^{2}$=0.29) and the FPC+M group (*p* = 0.03, ${{{\upeta}}}_{p}^{2}$=0.34); the remaining parameters were not significantly different. The specific results are shown in Fig. 5.

## Discussion

This study aimed to investigate the biomechanical characteristics of firefighters running while performing various rescue tasks to provide theoretical and practical guidance for firefighter training and injury prevention. The results revealed that while the FPC+GC and FPC+M rescue tasks exhibited a similar change pattern in gait parameters, their kinematic and kinetic parameters differed. Specifically, the FPC+GC group exhibited unchanged in most parameters, whereas the FPC+M group exhibited significant changes in the kinematic and kinetic parameters of the hip and knee joints. These findings suggest that the FPC+M rescue task may pose a higher risk of hip and knee injury than the FPC rescue task.

The FPC+GC rescue task changed firefighter gait performance. Park et al.^21^ on task loading in firefighters have shown that firefighters' gait changes significantly as the weight of the load increases: this is mainly reflected in the decrease in gait velocity and stride length and the increase in support time, which is consistent with the results of this study. In order to overcome the challenges of rescue tasks and ensure their safety^3,13^, firefighters chose a cautious gait strategy of sacrificing gait performance to improve stability when loading gas cans for rescue tasks^21^, which may be the main reason for the changes in firefighter gait parameters. This study also found that vGRF was significantly decreased in the FPC+GC rescue task compared to the FPC rescue task. Previous studies have suggested that significant changes in biomechanical parameters of the lower extremities have a greater correlation with gait velocity, and vGRF generally increases with increasing gait velocity^22,23^. In the present study, despite the FPC+GC group carrying a heavier load than the FPC group, their gait velocity decreased significantly, which may lead to a decrease in vGRF. On the other hand, this change could also be due to the firefighter's adaptations or changes in compensation strategies. Future studies could further explore the intrinsic mechanisms of vGRF alteration under controlled gait velocity conditions.

The FPC+M rescue task also changed the gait performance of firefighters. At the same time, kinetic parameters reflecting lower extremity injury risk were also changed. In terms of gait parameter changes, the results of this study showed that compared to the FPC rescue, the gait velocity and stride length of the FPC+M rescue task were significantly decreased, while the support time was significantly increased and compared to the FPC+GC rescue task, the support time of the FPC+M rescue task increased significantly. As with the FPC+GC rescue task, the firefighters in the FPC+M rescue task adopted a cautious gait strategy of sacrificing gait performance for stability as the load weight increased compared with the FPC rescue task^21^, which may be the main reason for the change in firefighter gait parameters. Unlike the FPC+GC rescue task, the FPC+M rescue task has a greater load weight. The increase in load weight significantly decreases the extension rate of the lower extremities^16^, which leads to the fact that the support time of the FPC+M rescue task is significantly increased than that of the FPC+GC rescue task in the results of this study. Interestingly, concerning kinetic parameters reflecting the risk of lower extremity injuries, this study also demonstrated a significant increase in hip extension moment and joint stiffness in the FPC+M group. The lower limbs have two crucial functions in the running stance period: maintaining stability and absorbing ground reaction force^16^. Cross et al.^24^ on weight-bearing running have shown that with the increase in load weight, participants would adopt a movement strategy of increasing the extension moment and joint work of the hip at the end of the stance to maintain stability and reduce ground reaction force. Although this movement strategy effectively increased hip power output to maintain running, it also inadvertently increased hip stiffness and risk of strain^25^. This may be the main reason for the increased extension moment and joint stiffness of the hip in the FPC+M group of this study.

Additionally, this study found that the knee joint's minimum flexion angle, stiffness, and maximum flexion moment were significantly increased for the FPC+M rescue task compared to the FPC and FPC+GC rescue tasks. Sell et al.^26^ have suggested that ACL shear and reaction forces increase as weight increases, increasing knee injury risk. In order to reduce the risk of injury during exercise, participants would adopt a self-protective regulatory mechanism, as evidenced by an increase in knee flexion angle and moment^26^, which is consistent with our findings. However, unlike the Sell et al.^26^ study, there was no significant change in knee extension moment in our study, which may lead to a lack of restraint in knee flexion, resulting in the risk of injuries. Future studies could further explore the underlying biomechanical mechanisms. Meanwhile, considering the results of this study on the kinetic parameters of the FPC+M rescue task, the strength training of firefighters' trunk, hip, and knee muscles should be strengthened in the future in the daily training, especially in the large weight training of 60 KG and above, in order to enhance the firefighters' hip and knee joint functions to reduce the risk of injuries in the real rescue tasks.

Trounson et al.'s^27^ study on the ankle joint under weight-bearing conditions have shown that participants adopt a movement strategy that decreases the ankle joint's plantarflexion angle as the load's weight increases. However, the results of this study showed that the ankle plantarflexion angle did not change as the loaded weight increased in the participants. It was analyzed that this may be more related to the firefighters' boots and the load weight. In the previous study, the maximum load weight was only 5% of the body weight, and the participants were wearing normal running shoes. However, in this study, the load weight was greater, and the firefighters' boots were made of unique materials. These boots could significantly limit the firefighters’ ROM of the ankle while protecting their safety^10^, which may be an important reason for the results of this study. In addition, the results of this study also showed that the extension and flexion moments of the hip, flexion moments of the knee, plantarflexion moments of the ankle, and peak flexion power of the hip were significantly increased in the FPC+M group compared to the FPC+GC group, which was significantly correlated with the increase in load weight. In general, weight-bearing running increases the load on the body, and the body needs to exert more force to maintain balance and provide running potential energy^28^. From the muscle perspective, participants increased the level of activation of the rectus femoris, biceps femoris, vastus lateralis, and gastrocnemius muscles^29^ to maintain balance and ensure the forward potential energy of running. In contrast, the increased activation level of lower limb joint-related muscle groups was the main reason for each joint's increased moment and power. Future studies could be combined with EMG and other testing methods for further analysis.

## Study limitation

There are some limitations to this study. First, valid data collection tests based on the limitations of the experimental conditions of one force platform may affect the normal running habits of firefighters, i.e., in order to quickly complete the experiment and thus adjust the running habits for the test, which may affect the results of this study in terms of gait parameters such as step width and stride length, as well as kinematics and kinetic parameters, and future studies should further validate the conclusions of this study. Second, we analyzed a limited number of biomechanical parameters, and future studies could also analyze the center of pressure and coronal plane's biomechanical parameters to investigate the effects of different rescue tasks on firefighter balance, rescue technique performance, and injury risk. Third, this study did not explore the rescue task characteristics of firefighters without FPCs and could be further investigated in future studies. Lastly, the passive marker motion analysis method was used in this study for biomechanical data collection. Despite the repeated measures design used in this study, the validity of the passive marker motion analysis method needs to be further validated under different varying conditions. Future studies could also use other data collection methods to validate the findings of this study.

## Conclusions

In both the FPC+GC and FPC+M rescue tasks, firefighters' gait performance was changed due to task loading, as evidenced by decreased gait velocity and stride length and increased support time. Compared with the FPC rescue task, from the perspective of injury risk, the FPC+GC rescue task does not increase the risk of lower limb injury for firefighters, but the FPC+M rescue task increases the risk of hip and knee injuries. Therefore, it is recommended that firefighters strengthen the strength training of trunk, hip and knee muscles under heavy loads (60 kg and above) in their daily training to reduce the risk of injuries during rescue tasks.

## Data Availability

The datasets used and/or analyzed during the current study available from the corresponding author on reasonable request.
